# Holding the frontline: a cross-sectional survey of emergency department staff well-being and psychological distress in the course of the COVID-19 outbreak

**DOI:** 10.1186/s12913-021-06555-5

**Published:** 2021-05-29

**Authors:** Gijs Hesselink, Lise Straten, Lars Gallée, Anne Brants, Joris Holkenborg, Dennis G. Barten, Yvonne Schoon

**Affiliations:** 1 Department of Emergency Medicine, Radboud Institute for Health Sciences, Radboud University Medical Center, Nijmegen, The Netherlands; 2 IQ healthcare, Radboud Institute for Health Sciences, Radboud University Medical Center, P.O. Box 9101, 114 IQ healthcare, 6500 HB Nijmegen, The Netherlands; 3 Department of Emergency Medicine, Canisius-Wilhelmina Hospital, Nijmegen, The Netherlands; 4 Department of Emergency Medicine, Rijnstate Hospital, Arnhem, The Netherlands; 5 Department of Emergency Medicine, VieCuri Medical Center, Venlo, The Netherlands; 6 Department of Geriatrics, Radboud Institute for Health Sciences, Radboud University Medical Center, Nijmegen, The Netherlands

**Keywords:** Coronavirus, COVID-19, Mental health, Well-being, Emergency department

## Abstract

**Background:**

The coronavirus disease 2019 (COVID-19) outbreak has been associated with stress and challenges for healthcare professionals, especially for those working in the front-line of treating COVID-19 patients. This study aimed to: 1) assess changes in well-being and perceived stress symptoms of Dutch emergency department (ED) staff in the course of the first COVID-19 wave, and 2) assess and explore stressors experienced by ED staff since the COVID-19 outbreak.

**Methods:**

We conducted a cross-sectional study. An online questionnaire was administered during June–July 2020 to physicians, nurses and non-clinical staff of four EDs in the Netherlands. Well-being and stress symptoms (i.e., cognitive, emotional and physical) were scored for the periods pre, during and after the first COVID-19 wave using the World Health Organization Well-Being Index (WHO-5) and a 10-point Likert scale. Stressors were assessed and explored by rating experiences with specific situations (i.e., frequency and intensity of distress) and in free-text narratives. Quantitative data were analyzed with descriptive statistics and generalized estimating equations (GEE). Narratives were analyzed thematically.

**Results:**

In total, 192 questionnaires were returned (39% response). Compared to pre-COVID-19, the mean WHO-5 index score (range: 0–100) decreased significantly with 14.1 points (*p* < 0.001) during the peak of the first wave and 3.7 points (< 0.001) after the first wave. Mean self-perceived stress symptom levels almost doubled during the peak of the first wave (≤0.005). Half of the respondents reported experiencing more moral distress in the ED since the COVID-19 outbreak. High levels of distress were primarily found in situations where the staff was unable to provide or facilitate necessary emotional support to a patient or family. Analysis of 51 free-texts revealed witnessing suffering, high work pressure, fear of contamination, inability to provide comfort and support, rapidly changing protocols regarding COVID-19 care and personal protection, and shortage of protection equipment as important stressors.

**Conclusions:**

The first COVID-19 wave took its toll on ED staff. Actions to limit drop-out and illness among staff resulting from psychological distress are vital to secure acute care for (non-)COVID-19 patients during future infection waves.

**Supplementary Information:**

The online version contains supplementary material available at 10.1186/s12913-021-06555-5.

## Background

On 30 January 2020, The World Health Organization declared the coronavirus disease (COVID-19) as a public health emergency of international concern [1]. By November 2020, more than fifty million confirmed COVID-19 cases [2] and 1.25 million deaths due to the coronavirus have been reported [3].

The COVID-19 outbreak has been associated with mental problems and challenges for many people, including healthcare professionals treating COVID-19 patients in the frontline [4–6]. Professionals were reported to have a high risk of experiencing mental health complaints, such as anxiety, stress, depression, sleep disturbance, loss of self-confidence, [7–9] as well as physical health complaints [9, 10]. Increased mental and physical health problems among healthcare staff in the midst of the pandemic can endanger the accessibility and quality of acute care. Psychological distress was reported to occur during previous virus outbreaks, and it contributed to the shortage of healthcare staff due to mental illness, sick leave or resignation [11–14]. Moreover, poor mental and physical health among staff might not only be detrimental to individuals but also may hinder professional performance and, in turn, the quality of care [15–17].

The impact of COVID-19 on healthcare professionals’ health status have been investigated in many previous studies [7–10]. However, they have not been adequately explored among staff working in the emergency department (ED); the most common entry point for acute hospital care. Better understanding of the impact of COVID-19 on the mental health of ED staff and insight into perceived stressors could help to identify appropriate psychosocial interventions ultimately aimed at securing continuous access and high-level quality of ED care for COVID-19 and ‘regular’ emergencies throughout the course of the pandemic. Therefore, this study has two aims: first, to assess changes in well-being and perceived stress symptoms of ED staff in the course of the first COVID-19 wave in the Netherlands; and second, to assess and explore the stressors experienced by ED staff since the COVID-19 outbreak.

## Methods

### Study design and setting

We performed a cross-sectional survey study in the period from June 18 to July 242,020. Quantitative and qualitative data were collected through an online questionnaire from clinical and non-clinical staff of four EDs (one academic and three regional teaching hospitals) in the eastern region of the Netherlands. On February 27, 2020, the first patient with COVID-19 was hospitalized in a Dutch hospital. This date marks the start of the COVID-19 outbreak in the Netherlands [18]. Since this date the number of hospitalized COVID-19 patients rapidly increased and reached first peak levels at the end of March/early April 2020. The first wave in the Netherlands ended early June 2020.

This study is reported in accordance with the STrengthening the Reporting of OBservational studies in Epidemiology (STROBE) guideline. The local ethics committee CMO region Arnhem - Nijmegen approved the study (registration number: 2020–7145). The survey started with an informed consent of the study. Participation was only possible after reading the informed consent and selecting the “agree” option, otherwise the questionnaire could not be filled out.

### Data collection

#### Participant recruitment and survey administration

All physicians (i.e., medical specialists and residents), nurses, nursing assistants and administrative staff (i.e., secretaries, administrative assistants, care coordinators) who worked in the ED since February 27, 2020, received a questionnaire. Eligible staff members were identified per study site by the head of the department or a senior staff member, and locally invited by e-mail to complete an online questionnaire. This email contained a standardized template consisting of general information about the study, how their personal data is stored and protected, and a link to the questionnaire. One reminder was sent 14 days after the initial invitation. In the invitation and reminder email staff members were kindly asked to fill in the questionnaire only once to avoid multiple responses from the same participant. Each participant completed the survey anonymously. Data were collected using Limesurvey – a frequently used and secured online questionnaire program – and subsequently transferred by GH to a secured database in the protected server of the Radboudumc.

#### Questionnaire

The questionnaire consisted of seven main sections: 1) general information on socio-demographics and occupation, 2) well-being, 3) stress symptoms, 4) moral stress; 5) experiences with stressors, 6) working climate and 7) managerial support. The data gathered on the last two sections were separately analysed in another study. General information included: gender, age, marital status, household size, caregiver tasks, professional function and hospital/study site, work experience and work-related information (i.e., contract hours, working overtime, treating COVID-19 patients). Well-being was evaluated using a Dutch version of the five-item version of the World Health Organization Well-Being Index (WHO-5) which has proven to be a valid screening tool for well-being [19]. The WHO-5 is a self-administered measure of well-being over a short time period, initially 2 weeks [20]. We asked participants to rate their well-being for three time periods: during the last 2 weeks *before* the COVID-19 outbreak (February 27), *during* the peak of the first COVID-19 wave (February 27 to April 1) and *after* the first COVID-19 wave (the last 2 weeks since receiving the questionnaire). In the Netherlands, the first COVID-19 wave came to an end in the beginning of June [18]. The WHO-5 consists of five positively worded items that are rated on a 6-point Likert scale, ranging from 0 (at no time) to 5 (all of the time).

Stress symptoms were evaluated for the abovementioned three time periods on a Likert scale ranging from 0 (none) to 10 (very much). We asked participants to rate symptoms related to cognitive stress (i.e., concentration problems, constant worrying, poor judgement, forgetfulness), emotional stress (i.e., anxiety, agitation, moodiness, irritability, anger, feeling overwhelmed) and physical stress (i.e., aches, pains, nausea, dizziness, rapid heart rate, hyperventilation, sleeplessness) [21]. In addition, participants were asked if they experienced more, less or the same level of moral distress since the outbreak of the COVID-19 outbreak. Moral distress has been defined as a consequence when someone knows what is ethically right but for different reasons cannot act accordingly [22, 23].

Levels of distress were assessed by rating 13 items describing specific clinical situations found to generate distress among health care staff (i.e., stressors). Participants were asked to rate, on a 5-point Likert scale, how often they experienced this situation (frequency; never-very often) and how much the situation would disturb them (intensity; not at all-very much) since the COVID-19 outbreak. Five situations were derived from the Measure of Moral Distress for Healthcare Professionals (MMD-HP) [24]. Eight situations were formulated based on findings from previous studies evaluating stress reactions by healthcare professionals during previous virus outbreaks, [12, 13] and the experiences with COVID-19 in the ED shared by colleagues. In addition, participants were invited to describe, in free-text fields, a maximum of three stressors (i.e., a specific situation or repeated factor causing distress) they had experienced in the ED since the COVID-19 outbreak.

### Data analysis

All statistical analyses were performed using SPSS version 23.0. Descriptive statistics were used to summarize respondent’s sociodemographic and occupational characteristics, well-being, stress symptom levels, changes in moral distress, and the frequency and intensity of stressors. Frequency, percentage, mean, standard deviation (SD), median and interquartile range (IQR) scores were used according to the data type.

The raw scores on the WHO-5 items were transformed to an index score between 0 to 100, with lower scores indicating worse well-being. An index score of ≤50 indicates poor well-being and suggests further investigation into possible symptoms of depression [20]. Cronbach’s alpha coefficients for the WHO-5 in this study were respectively 0.90 (before the COVID-19 outbreak), 0.89 (during the peak of the first COVID-19 wave) and 0.91 (after the first COVID-19 wave). After checking WHO-5 index scores and stress symptom levels for normality, we performed generalized estimating equations (GEE) to model the development of well-being and stress symptom levels over time. We used a linear model with the outcome as a dependent variable and time as an independent factor. The GEE model took into account the correlations between repeated measurements within the same subject. An exchangeable correlation matrix was used, which means that the observations within a subject are assumed to be equally correlated. Five cases had missing values on at least one of the stress symptom variables and were excluded from the analysis. In addition, we used the Pearson’s Chi-squared test for the comparison of reported changes in moral distress per type of profession. A *p* value <.05 was considered to be statistically significant, based on two-sided testing. Differences (pre, during and post) in well-being and stress symptom levels were investigated across gender groups (male versus female) and types of professional function (i.e., physicians and nurses versus the rest of the respondents) using the unpaired t-test.

A mean composite distress score was calculated for each of the 13 described situations to compare levels of distress by situation and participant type. A composite distress score was calculated by multiplying frequency and intensity (0–16) with higher scores suggesting higher levels of distress. The level of distress experienced is a function of how often a situation occurs and how distressing it is when experienced [24]. The classification of low, moderate and high scores (frequency, intensity and composite score) were based on the percentiles of the means of the 13 items as shown in Additional file 1. Cases with missing values were excluded from the analysis. Additional stressors described by respondents in the free-text fields were thematically analysed in Microsoft Excel using inductive and deductive reasoning. First, two researchers (LS and GH) independently read all text fragments and inductively identified overarching themes. The final set of themes was established after discussion between both researchers. Subsequently, each description of a stressor was deductively placed under one of the identified themes, which resulted in an overview of described stressors organized per theme. For each theme, one or two text fragments were selected to illustrate experienced types of stressors.

## Results

### Respondent characteristics

A total of 495 eligible persons were approached to participate. One hundred ninety-two completed questionnaires were returned, resulting in a response rate of 39%. Respondents were comparable to the total population eligible to participate when looking at the gender ratio, professional function and age. A comparison is shown in Additional file 2.

Data on the characteristics of the respondents are shown in Table 1. Of the 192 respondents, 140 (72.9%) were female. Respondents had a mean age of 39.6 (SD 11.8) years. More than half (54.2%) of the respondents were nurses. The majority (83.3%) was structurally employed in the ED and treated COVID-19 patients (87.0%). A large group of respondents (40.1%) reported that their number of work shifts had increased since the COVID-19 outbreak. Of the 164 responses, 82 (50%) respondents reported to experience more moral distress in the ED since the COVID-19 outbreak. Most of these respondents were nurses (56%; < 0.001).

**Table 1:** Characteristics of respondents

	Total (***n*** = 192)	ED 1 (***n*** = 63)	ED 2 (***n*** = 39)	ED 3 (***n*** = 25)	ED 4 (***n*** = 65)
Gender, n (%)					
*Male*	52 (27.1)	22 (34.9)	9 (23.1)	5 (20.0)	16 (24.6)
*Female*	140 (72.9)	41 (65.1)	30 (76.9)	20 (80.0)	49 (75.4)
Age in years, mean (SD)	39.6 (11.8)	41.5 (12.7)	40.1 (11.3)	41.4 (10.2)	36.9 (11.5)
Professional function					
*Nurse, n (%)*	104 (54.2)	33 (52.4)	24 (61.5)	13 (52.0)	31 (47.7)
*Physician*^a^*, n (%)*	53 (27.6)	14 (22.2)	9 (23.1)	6 (24.0)	24 (36.9)
*Administrative staff*^b^*, n(%)*	25 (13.0)	9 (14.3)	5 (12.8)	6 (24.0)	0 (0)
*Nursing assistants, n (%)*	10 (5.2)	1 (1.6)	1 (2.6)	0 (0)	8 (12.3)
Employed in the emergency department					
*Yes*	160 (83.3)	55 (87,3)	38 (97,4)	25 (100,0)	42 (64,6)
*No*	32 (16.7)	8 (12.7)	1 (2.6)	0 (0)	23 (35.4)
Years of work experience in current function, mean (SD)	7.1 (7.3)	7.8 (7.7)	7.9 (8.2)	6.8 (7.3)	6.0 (6.1)
Working hours per week, mean (SD)	31.4 (8.8)	30.9 (9.8)	29.6 (5.9)	29.5 (6.1)	33.7 (9.8)
Work shifts since the first COVID-19 outbreak^c^					
*Increased, n (%)*	77 (40.1)	33 (52.4)	13 (33.3)	13 (52.0)	18 (27.7)
*Decreased, n (%)*	6 (3.1)	2 (3.2)	1 (2.6)	0 (0)	3 (4.6)
*No changes, n (%)*	109 (56.8)	28 (44.4)	25 (64.1)	12 (48.0)	44 (67.7)
Treated COVID-19 patients, n (%)	167 (87.0)	48 (76.2)	34 (87.2)	23 (92.0)	62 (95.4)

^a^Medical specialists and residents

^b^Secretary, administrative support, care coordinators

^c^Marked by February 27, 2020 (on this date, the first COVID-19 patient was hospitalized in the Netherlands)

### Changes in well-being

**Fig. 1:**
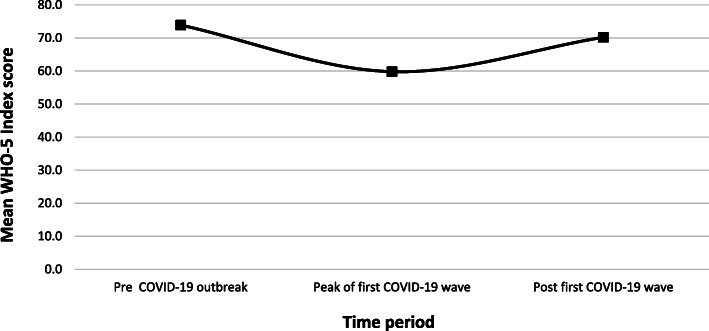
Development of respondents’ well-being in the course of the COVID-19 outbreak

Differences were found in the mean WHO-5 index scores before, during and after the first COVID-19 wave for all respondents combined (Table 2). Compared to baseline (i.e., during the last 2 weeks before the COVID-19 outbreak), the mean WHO-5 index score decreased significantly with 14.1 points (*p* < 0.001) during the peak of the first wave and 3.7 points (*p* < 0.001) after the first wave. On average, respondents’ well-being improved after the first COVID-19 wave, but remained lower than before the COVID-19 outbreak. The development of respondents’ well-being in the course of the COVID-19 outbreak is shown in Fig. 1. Additional file 3 shows the developments of well-being per professional function and gender group. No statistically significant differences in mean WHO-5 index scores (before, during and after the first COVID-19 wave) were found across gender groups. The mean WHO-5 index score was significantly higher for physicians compared to the rest of the respondents during the peak of the first wave (*p* < 0.01).

**Table 2:** Longitudinal modelling on respondent’s WHO-5 index scores (*n* = 192)

	Mean (SD)	Beta coefficient	SE	95% CI	***p***
Period					< 0.001
*Pre COVID-19 outbreak*	73.9 (13.9)	Reference			
*Peak of first COVID-19 wave*	59.8 (18.6)	−14.1	1.2	−16.6 to −11.7	< 0.001
*Post first COVID-19 wave*	70.2 (16.6)	−3.7	1.0	−5.6 to −1.7	< 0.001

*SD* Standard Deviation, *SE* Standard Error, *CI* Confidence Interval.

### Changes in self-reported stress symptoms

**Fig. 2:**
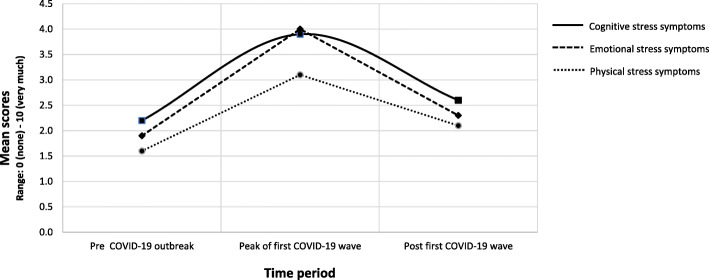
Development of respondents’ stress symptoms in the course of the COVID-19 outbreak

Differences were also found in the mean scores on self-perceived stress symptoms (Table 3). Compared to baseline, the mean score on cognitive stress symptoms increased significantly with 1.7 points (*p* < 0.001) during the peak of the first wave, and 0.4 points (=0.005) after the first wave. Emotional stress symptoms increased significantly with 2.1 points (*p* < 0.001) and 0.4 points (=0.003), respectively. Physical stress symptoms increased significantly with 1.5 points (*p* < 0.001) and 0.4 points (< 0.001), respectively. On average, experienced stress symptoms almost doubled during the peak of the first wave. Stress symptom levels decreased after the first COVID-19 wave, but they remained higher than before the COVID-19 outbreak. Figure 2 illustrates the development of respondents’ stress symptoms in the course of the COVID-19 outbreak. Additional file 4 shows the developments of stress symptoms per professional function and gender group. No statistically significant differences in self-perceived stress symptoms were found across gender groups, except for a higher mean score for males on emotional stress symptoms before the outbreak (*p* = 0.01). Compared to the rest of the respondents, physicians scored significantly lower on self-perceived emotional and physical stress symptoms during the peak of the first wave (*p* = 0.04 and *p* < 0.01 respectively).

**Table 3:** Longitudinal modelling on stress symptom scores (*n* = 187)

	Mean (SD)	Beta coefficient	SE	95% CI	***p***
**Cognitive stress symptoms**					< 0.001
Pre COVID-19 outbreak	2.2 (1.8)	Reference			
Peak of first COVID-19 wave	3.9 (2.5)	1.7	0.15	1.4 to 2.0	< 0.001
Post first COVID-19 wave	2.6 (2.4)	0.4	0.13	0.1 to 0.6	0.005
**Emotional stress symptoms**					< 0.001
Pre COVID-19 outbreak	1.9 (1.8)	Reference			
Peak of first COVID-19 wave	4.0 (2.6)	2.1	0.2	1.7 to 2.4	< 0.001
Post first COVID-19 wave	2.3 (2.3)	0.4	0.1	0.1 to 0.7	0.003
**Physical stress symptoms**					< 0.001
Pre COVID-19 outbreak	1.6 (1.8)	Reference			
Peak of first COVID-19 wave	3.1 (2.7)	1.5	0.2	1.2 to 1.8	< 0.001
Post first COVID-19 wave	2.1 (2.3)	0.4	0.1	0.2 to 0.7	< 0.001

*SD* Standard Deviation, *SE* Standard Error, *CI* Confidence Interval

### Levels of distress on specific clinical situations since the COVID-19 outbreak

**Fig. 3:**
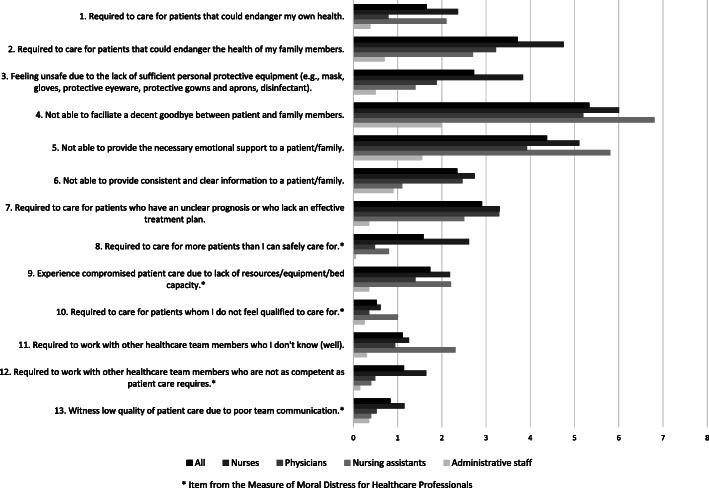
Mean composite distress score, by profession and overall

High levels of distress (i.e., composite score) were found on three of the thirteen situations, namely situations where respondents were: not able to facilitate a decent goodbye between patient and family members (item 4), not able to provide the necessary emotional support to a patient and family members (item 5), and required to care for patients that could endanger the health of their own family (item 2). Figure 3 shows the mean composite distress score for each item, by profession and overall. High scores on frequency and distress intensity were also found for the same three situations (see Additional files 5, 6 and 7). Moderate scores were found for eight situations, with highest scores in this group for: feeling unsafe due to the lack of sufficient personal protective equipment (item 3) and not being able to provide consistent and clear information to a patient or family members (item 6). None of the situations scored high on frequency *and* moderate-low on intensity, or vice versa. Highest distress scores were mostly found for nurses (eight situations) followed by nursing assistants (five situations).

### Stressors described by respondents since the COVID-19 outbreak

Respondents described 51 stressors which were organized into six themes (Table 4). Most narratives related to witnessing patients’ and family members’ suffering (31%). The rapid health deterioration and the anxiety of patients with COVID-19, and the reactions of family members had a significant emotional impact on respondents. Nineteen percent related to increased work pressure resulting from COVID-19 care. Additional precaution measures and the preparation time needed to care for an increasing number of COVID-19 patients required the staff to work extra hard under difficult circumstances. Seventeen percent of the described situations referred to the respondents’ fear of being contaminated with the coronavirus in the ED and, in turn, fear of contaminating others. The other narratives were related to: the inability to comfort or support patients and relatives mainly because of protection measures (10%), rapidly changing protocols regarding COVID-19 care and personal protection (10%); and the shortage of high-quality personal protection equipment in the ED (10%).

**Table 4:** Stressors described by respondents since the COVID-19 outbreak

Themes	N (%)	Quotes
Witnessing patients and family suffering	16 (30.8)	*“Patients arriving by ambulance and gasping for air, with mottled skin, actively dying. With only one person allowed to accompany him. That’s emotionally heavy. Inhumane”.*
		*“Seeing family members leaving the ED not knowing if they would ever see their loved ones alive again”.*
Work pressure	10 (19.2)	*“Caring for COVID-19 patients in addition to regular acute care with less professionals”.*
		*“Not able to fall back on the support of colleagues because of the isolation procedure”.*
Fear of contamination	9 (17.3)	*“Patient has already been in the ED for four hours without being suspected of having COVID-19. Suddenly, just before hospital admission, the doctor decides to perform a COVID-19 test. So, I was not protected during these hours”.*
		*“The staff canteen is crowded with personnel without any social distancing”.*
Inability to provide comfort and support	5 (9.6)	*“Telling people at the counter that only one person or no people are allowed to accompany their loved one. That’s heart-breaking”.*
		*“Not being able to guide family members in saying goodbye to a patient. Communication through the use of an iPad is not very personal, especially when you wear a protection mask”.*
Changing protocols regarding COVID-19 care and personal protection	5 (9.6)	*“Protocols were constantly revised, sometimes multiple times a day”.*
Shortage of high-quality PPE	5 (9.6)	*“PPE’s were scarce at times and depended on availability. This affected the quality of care: less contacts with patients, less supervision,* et cetera*”.*

*ED* Emergency Department, *PPE* Personal Protection Equipment

## Discussion

The COVID-19 outbreak had a serious impact on the well-being and mental health of ED staff. In the peak of the first wave the staff well-being dropped to a level that is close to the threshold (≤50) indicating the need for screening for depression [20]. Cognitive, emotional and physical stress symptoms almost doubled. These findings are in line with outcomes of recent meta-analyses indicating the psychological impact of COVID-19 on healthcare providers [4–6]. Several recent studies have demonstrated the psychological impact that COVID-19 particularly has on professionals working in emergency departments, [25–27] and in other frontlines such as the Intensive Care Unit [7–10, 28, 29]. Although well-being and the stress symptom levels increased after the first wave, they did not meet the reported levels before the COVID-19 outbreak. This difference may be explained by the staff mentally recovering from the first wave at the time the survey was administered. It is worrisome to notice that the ED staff did not have much time to recover given the constant flow of COVID-19 admissions and the rapid arrival of new contamination waves. Furthermore, we found lower levels of well-being and higher levels of perceived stress symptoms among nurses and supporting staff compared to physicians, which may be partly explained by nurses having closer and prolonged contact with patients compared to physicians [4].

The COVID-19 outbreak also had an impact on the psychological distress of the ED staff. A large part of the ED staff (50%) experienced more moral distress since the outbreak. Our findings showed high levels of distress, both in frequency and intensity, which were primarily caused by situations where the staff was hindered in providing the clinical and emotional care they are used to provide under ‘normal’ conditions. The use of self-evident safety precautions regarding the virus and the limited options to effectively treat COVID-19 is presumably disturbing to many professionals as they are in sharp contrast with perceived standards of good care. The fear of being exposed to COVID-19 at work and taking the infection home to their family was reported as another important stressor, also known from previous studies [10, 30, 31]. Especially nurses in this study were found to experience distress the most, which may be once again be explained by their role as the primary care giver and contact person for patients and family members in the ED. This corresponds with the findings of a recent meta-analysis on the psychological impact of COVID-19 on healthcare workers, [4] and findings from similar studies on the severe acute respiratory syndrome (SARS) outbreak almost a decade ago, [12, 32, 33].

Respondents’ narratives revealed multiple factors causing psychological distress. Many of these factors are potentially amendable to managerial intervention. For example, by managers encouraging staff to ask for help when they need it and by offering various types of support (e.g. mental coaching, peer support and an online training module) [34–36]. Healthcare professionals are often self-reliant and many do not ask for help. However, this behavioural mechanism may not serve them well in a time of burgeoning workload in difficult and emotional circumstances. Moreover, a dedicated, screen-free place could help staff to destress and recuperate [37, 38]. Adjustments to the ED’s physical environment to facilitate social distancing and separating COVID-19 care from regular acute care could contribute to a safer and less stressful work environment. The use of mobile devices and applications could assist ED staff in finding ways to facilitate personal contact and to provide emotional support to COVID-19 patients and their family members. Finally, the provision of sufficient and appropriate personal protective equipment, and work rotation schedules which enable sufficient rest in the face of this ongoing pandemic seem paramount [35, 36].

To our knowledge, this study is one of the first to investigate the impact of COVID-19 on the well-being and phycological distress of various types of professionals working in multiple EDs. Several limitations of the study should however be noted. First, the response rate to our questionnaire was modest at 39%. Unfortunately, web-based survey research among healthcare professionals shows substantial variation in reported response rates and rates below 20% are not uncommon [39–41]. Our response rate might increase the possibility of non-response bias and under-representation of specific groups. Moreover, there is a possibility of self-selection bias where ED professionals that relate to the topics of well-being and psychological distress respond at higher rates. For reasons of confidentiality, we were unable to distinct responders from non-responders and compare both groups on statistical differences. The respondents were nevertheless comparable to the total population eligible to participate when looking at the gender ratio, professional function and age. Moreover, if respondents do not significantly differ from non-respondents with respect to the relevant characteristics, a low response rate is not necessarily associated with inferior data [39]. Second, well-being and stress symptoms were retrospectively reported by respondents over three time periods, with the first period several months ago. This may have led to recall bias and less reliable scores. Third, changes in stress symptoms were based on self-reported estimations using single-item measures that were only tested on face validity. We deliberately opted for these single items to minimize respondent burden in the midst of a pandemic. However, apart from the risk of response bias, which is commonly associated with self-reported data, [42] the use of these single items might have been insufficient in adequately capturing our constructs of interest. Fourth, our sample size consisted of staff from four EDs in one country. Although, the COVID-19 pandemic seriously affects health systems and professionals around the globe, our findings may not be representative at the international level as the demand for acute COVID-19 care and available resources may differ per country and in time.

## Conclusions

This study adds to recent literature showing that he COVID-19 pandemic takes its toll on ED staff. The question is how long the frontline remains intact with the ongoing pandemic. We hope that this study contributes to a public awareness of the impact of infection peaks on ED staff. Timely and adequate actions by ED and hospital management to limit drop-out and illness among staff resulting from psychological distress are vital to secure acute care for (non)COVID-19 patients during future infection waves.

## Supplementary Information

**Additional file 1: Table S1.** The classification of distress composite, frequency and intensity scores into low, moderate and high.

**Additional file 2: Table S2.** Respondents compared to all eligible persons approached for study participation on gender, age and professional function.

**Additional file 3: Figure S1.** Longitudinal modelling on mean WHO-5 index scores per professional function. **Figure S2.** Longitudinal modelling on mean WHO-5 index scores per gender group.

**Additional file 4: Figure S1.** Longitudinal modelling on mean cognitive stress symptom scores per professional function. **Figure S2.** Longitudinal modelling on mean cognitive stress symptom scores per gender group. **Figure S3.** Longitudinal modelling on mean emotional stress symptom scores per professional function. **Figure S4.** Longitudinal modelling on mean emotional stress symptom scores per gender group. **Figure S5.** Longitudinal modelling on mean physical stress symptom scores per professional function. **Figure S6.** Longitudinal modelling on mean physical stress symptom scores per gender group.

**Additional file 5.** Table. Composite distress score, frequency and intensity of distress on thirteen specific clinical situations, overall and by profession.

**Additional file 6.** Figure. Mean frequency score, overall and by profession.

**Additional file 7.** Figure. Mean intensity score, overall and by profession.

## Data Availability

Deidentified datasets are available from the corresponding author on reasonable request.

## Abbreviations

COVID-19: Coronavirus Disease 2019
ED: Emergency Department
WHO-5: World Health Organization Well-Being Index
GEE: Generalized Estimating Equations
STROBE: STrengthening the Reporting of OBservational studies in Epidemiology
MMD-HP: Measure of Moral Distress for Healthcare Professionals
SD: Standard Deviation
IQR: Interquartile Range
SE: Standard Error
CI: Confidence Interval
PPE: Personal Protection Equipment
