# Supplementary material for: Holding the frontline: a cross-sectional survey of emergency department staff well-being and psychological distress in the course of the COVID-19 outbreak
Source: BMC Health Serv Res. 2021 May 29;21:525. doi: 10.1186/s12913-021-06555-5 (PMC8164246; doi:10.1186/s12913-021-06555-5)
Supplement: Supplementary file 1 — Additional file 1: Table S1. The classification of distress composite, frequency and intensity scores into low, moderate and high. [file 12913_2021_6555_MOESM1_ESM.docx]

| **S1 Table. The classification of distress composite, frequency and intensity scores into low, moderate and high** | | | |
| --- | --- | --- | --- |
|  | **Low (25% percentile)** | **Moderate** | **High (75% percentile)** |
| Composite score, mean | <1.13 | 1.13-3.31 | >3.31 |
| Frequency, mean | <0.72 | 0.72-1.63 | >1.63 |
| Intensity, mean | <0.62 | 0.62-1.47 | >1.47 |
