# Supplementary material for: Holding the frontline: a cross-sectional survey of emergency department staff well-being and psychological distress in the course of the COVID-19 outbreak
Source: BMC Health Serv Res. 2021 May 29;21:525. doi: 10.1186/s12913-021-06555-5 (PMC8164246; doi:10.1186/s12913-021-06555-5)
Supplement: Supplementary file 2 — Additional file 2: Table S2. Respondents compared to all eligible persons approached for study participation on gender, age and professional function. [file 12913_2021_6555_MOESM2_ESM.docx]

| **S2 Table. Respondents compared to all eligible persons approached for study participation on gender, age and professional function** | | | | | | | | | | |
| --- | --- | --- | --- | --- | --- | --- | --- | --- | --- | --- |
|  | **Respondents** | | | | | **Eligible persons approached for study participation** | | | | |
|  | **Total (n=192)** | **ED 1**  **(n=63)** | **ED 2 (n=39)** | **ED 3 (n=25)** | **ED 4 (n=65)** | **Total (n=426)** | **ED 1**  **(n=100)** | **ED 2 (n=93)** | **ED 3 (n=115)** | **ED 4**  **(n=118)** |
| Gender, n (%) |  |  |  |  |  |  |  |  |  |  |
| *Male* | 52 (27.1) | 22 (34.9) | 9 (23.1) | 5 (20.0) | 16 (24.6) | 128 (30.0) | 38 (38.0) | 27 (29.0) | 31 (27.0) | 32 (27.1) |
| *Female* | 140 (72.9) | 41 (65.1) | 30 (76.9) | 20 (80.0) | 49 (75.4) | 298 (70.0) | 62 (62.0) | 66 (71.0) | 84 (73.0) | 86 (72.9) |
| Age in years, mean (SD) | 39.6 (11.8) | 41.5 (12.7) | 40.1 (11.3) | 41.4 (10.2) | 36.9 (11.5) | 39.2 (11.2) | 42.9 (11.5)ǂ | 39.2 (11.1) | 38.0 (9.5) | 37.2 (11.8)ǂ |
| Professional function |  |  |  |  |  |  |  |  |  |  |
| *Nurse, n (%)* | 104 (54.2) | 33 (52.4) | 24 (61.5) | 13 (52.0) | 31 (47.7) | 234 (54.8) | 61 (60.4) | 54 (58.1) | 62 (53.9) | 57 (48.3) |
| *Physician*, n (%)* | 53 (27.6) | 14 (22.2) | 9 (23.1) | 6 (24.0) | 24 (36.9) | 118 (27.6) | 16 (15.8) | 23 (24.7) | 34 (29.6) | 45 (38.1) |
| *Administrative staff†, n(%)* | 25 (13.0) | 9 (14.3) | 5 (12.8) | 6 (24.0) | 0 (0) | 48 (11.2) | 16 (25.8) | 11 (11.8) | 19 (16.5) | 2 (1.7) |
| *Nursing assistants, n (%)* | 10 (5.2) | 1 (1.6) | 1 (2.6) | 0 (0) | 8 (12.3) | 26 (6.1) | 7 (6.9) | 5 (5.4) | 0 (0) | 14 (11.9) |
| *Medical specialists and residents.  †Secretaries, administrative support, team managers, care coordinators and heads of department.  ǂ 9 missing values. | | | | | | | | | | |
