# Supplementary material for: Holding the frontline: a cross-sectional survey of emergency department staff well-being and psychological distress in the course of the COVID-19 outbreak
Source: BMC Health Serv Res. 2021 May 29;21:525. doi: 10.1186/s12913-021-06555-5 (PMC8164246; doi:10.1186/s12913-021-06555-5)
Supplement: Supplementary file 3 — Additional file 3: Figure S1. Longitudinal modelling on mean WHO-5 index scores per professional function. Figure S2. Longitudinal modelling on mean WHO-5 index scores per gender group. [file 12913_2021_6555_MOESM3_ESM.docx]

**S3 Figure 1. Longitudinal modelling on mean WHO-5 index scores per professional function**

**S3 Figure 2. Longitudinal modelling on mean WHO-5 index scores per gender group**
