# Supplementary material for: Holding the frontline: a cross-sectional survey of emergency department staff well-being and psychological distress in the course of the COVID-19 outbreak
Source: BMC Health Serv Res. 2021 May 29;21:525. doi: 10.1186/s12913-021-06555-5 (PMC8164246; doi:10.1186/s12913-021-06555-5)
Supplement: Supplementary file 4 — Additional file 4: Figure S1. Longitudinal modelling on mean cognitive stress symptom scores per professional function. Figure S2. Longitudinal modelling on mean cognitive stress symptom scores per gender group. Figure S3. Longitudinal modelling on mean emotional stress symptom scores per professional function. Figure S4. Longitudinal modelling on mean emotional stress symptom scores per gender group. Figure S5. Longitudinal modelling on mean physical stress symptom scores per professional function. Figure S6. Longitudinal modelling on mean physical stress symptom scores per gender group. [file 12913_2021_6555_MOESM4_ESM.docx]

**S4 Figure 1. Longitudinal modelling on mean cognitive stress symptom scores per professional function**

**S4 Figure 2. Longitudinal modelling on mean cognitive stress symptom scores per gender group**

**S4 Figure 3. Longitudinal modelling on mean emotional stress symptom scores per professional function**

**S4 Figure 4. Longitudinal modelling on mean emotional stress symptom scores per gender group**

**S4 Figure 5. Longitudinal modelling on mean physical stress symptom scores per professional function**

**S4 Figure 6. Longitudinal modelling on mean physical stress symptom scores per gender group**
