# Supplementary material for: Holding the frontline: a cross-sectional survey of emergency department staff well-being and psychological distress in the course of the COVID-19 outbreak
Source: BMC Health Serv Res. 2021 May 29;21:525. doi: 10.1186/s12913-021-06555-5 (PMC8164246; doi:10.1186/s12913-021-06555-5)
Supplement: Supplementary file 5 — Additional file 5. Table. Composite distress score, frequency and intensity of distress on thirteen specific clinical situations, overall and by profession. [file 12913_2021_6555_MOESM5_ESM.docx]

| **S5 Table: Composite distress score, frequency and intensity of distress on thirteen specific clinical situations, overall and by profession** | | | | | |
| --- | --- | --- | --- | --- | --- |
|  | **Total (n=167)** | **Physicians (n=48)** | **Nurses (n=90)** | **Nursing Assistants**  **(n=10)** | **Administrative Staff**  **(n=20)** |
| 1. Required to care for patients that could endanger my own health. |  |  |  |  |  |
| *Composite, mean (sd)* | 1.65 (2.49) | 0.79 (1.15) | 2.36 (2.98)* | 2.10 (2.13) | 0.38 (1.36)* |
| *Composite, median (range)* | 0 (0-12) | 0 (0-4) | 1 (0-12) | 2 (0-6) | 0 (0-6) |
| *Frequency, mean (sd)* | 0.76 (0.92) | 0.60 (0.79) | 0.97 (1.00) | 0.90 (0.88) | 0.14 (0.48) |
| *Frequency, median (range)* | 0 (0-3) | 0 (0-2) | 1 (0-3) | 1 (0-2) | 0 (0-2) |
| *Intensity, mean (sd)* | 1.42 (1.07) | 1.00 (0.83) | 1.84 (1.00) | 1.40 (1.27) | 0.62 (0.97) |
| *Intensity, median (range)* | 1 (0-4) | 1 (0-3) | 2 (0-4) | 2 (0-3) | 0 (0-3) |
| 2. Required to care for patients that could endanger the health of my family members. |  |  |  |  |  |
| *Composite, mean (sd)* | 3.71 (3.99) | 3.22 (3.44)* | 4.75 (4.27)* | 2.70 (3.30) | 0.70 (2.05)* |
| *Composite, median (range)* | 2 (0-16) | 2 (0-12) | 4 (0-16) | 1 (0-9) | 0 (0-9) |
| *Frequency, mean (sd)* | 1.65 (1.26) | 1.65 (1.16) | 1.98 (1.20) | 1.30 (1.34) | 0.35 (0.81) |
| *Frequency, median (range)* | 1 (0-4) | 2 (0-4) | 2 (0-4) | 1 (0-3) | 0 (0-3) |
| *Intensity, mean (sd)* | 1.51 (1.16) | 1.38 (1.10) | 1.90 (1.06) | 1.10 (1.10) | 0.35 (0.81) |
| *Intensity, median (range)* | 2 (0-4) | 1 (0-3) | 2 (0-4) | 1 (0-3) | 0 (0-3) |
| 3. Feeling unsafe due to the lack of sufficient personal protective equipment (e.g., mask, gloves, protective eyeware, protective gowns and aprons, disinfectant). |  |  |  |  |  |
| *Composite, mean (sd)* | 2.72 (3.71) | 1.88 (2.43) | 3.83 (4.32)* | 1.40 (1.84) | 0.50 (2.01)* |
| *Composite, median (range)* | 1 (0-16) | 1 (0-9) | 2 (0-16) | 1 (0-6) | 0 (0-9) |
| *Frequency, mean (sd)* | 1.21 (1.18) | 1.02 (0.98) | 1.58 (1.24) | 0.80 (0.79) | 0.20 (0.70) |
| *Frequency, median (range)* | 1 (0-4) | 1 (0-3) | 1 (0-4) | 1 (0-2) | 0 (0-3) |
| *Intensity, mean (sd)* | 1.23 (1.19) | 1.02 (1.00) | 1.60 (1.24) | 1.10 (0.99) | 0.20 (0.70) |
| *Intensity, median (range)* | 1 (0-4) | 1 (0-3) | 1 (0-4) | 1 (0-3) | 0 (0-3) |
| 4. Not able to faciliate a decent goodbye between patient and family members. |  |  |  |  |  |
| *Composite, mean (sd)* | 5.33 (4.59) | 5.19 (4.73) | 6.00 (4.44)* | 6.80 (5.73) | 2.00 (2.66)* |
| *Composite, median (range)* | 4 (0-16) | 4 (0-16) | 6 (0-16) | 7.5 (0-16) | 0 (0-6) |
| *Frequency, mean (sd)* | 1.88 (1.28) | 1.88 (1.33) | 2.07 (1.15) | 2.30 (1.70) | 0.85 (1.04) |
| *Frequency, median (range)* | 2 (0-4) | 2 (0-4) | 2 (0-4) | 3 (0-4) | 0 (0-3) |
| *Intensity, mean (sd)* | 2.11 (1.36) | 2.10 (1.36) | 2.39 (1.25) | 2.00 (1.49) | 1.00 (1.26) |
| *Intensity, median (range)* | 2 (0-4) | 2 (0-4) | 3 (0-4) | 2.5 (0-4) | 0 (0-3) |
| 5. Not able to provide the necessary emotional support to a patient/family. |  |  |  |  |  |
| *Composite, mean (sd)* | 4.37 (4.29) | 3.92 (4.28) | 5.10 (4.29)* | 5.80 (5.14) | 1.55 (2.28)* |
| *Composite, median (range)* | 4 (0-16) | 2 (0-16) | 4 (0-16) | 4 (0-16) | 0.5 (0-8) |
| *Frequency, mean (sd)* | 1.67 (1.19) | 1.54 (1.22) | 1.90 (1.10) | 2.10 (1.45) | 0.75 (0.91) |
| *Frequency, median (range)* | 2 (0-4) | 1 (0-4) | 2 (0-4) | 2 (0-4) | 0.5 (0-3) |
| *Intensity, mean (sd)* | 1.89 (1.25) | 1.79 (1.27) | 2.14 (1.18) | 2.10 (1.29) | 0.95 (1.15) |
| *Intensity, median (range)* | 2 (0-4) | 2 (0-4) | 2 (0-4) | 2 (0-4) | 0.5 (0-4) |
| 6. Not able to provide consistent and clear information to a patient/family. |  |  |  |  |  |
| *Composite, mean (sd)* | 2.34 (3.21) | 2.46 (3.30) | 2.74 (3.44) | 1.10 (1.00) | 0.90 (1.97)* |
| *Composite, median (range)* | 1 (0-16) | 1 (0-12) | 1 (0-16) | 1 (0-2) | 0 (0-6) |
| *Frequency, mean (sd)* | 1.18 (1.07) | 1.19 (1.07) | 1.37 (1.09) | 0.90 (0.74) | 0.45 (0.89) |
| *Frequency, median (range)* | 1 (0-4) | 1 (0-4) | 1 (0-4) | 1 (0-3) | 0 (0-3) |
| *Intensity, mean (sd)* | 1.18 (1.16) | 1.35 (1.26) | 1.28 (1.12) | 0.90 (0.88) | 0.45 (0.89) |
| *Intensity, median (range)* | 1 (0-4) | 1 (0-4) | 2 (0-4) | 2 (0-4) | 0 (0-3) |
| 7. Required to care for patients who have an unclear prognosis or who lack an effective treatment plan. |  |  |  |  |  |
| *Composite, mean (sd)* | 2.90 (3.43) | 3.29 (3.76) | 3.30 (3.31)* | 2.50 (3.89) | 0.35 (1.35)* |
| *Composite, median (range)* | 2 (0-16) | 2 (0-16) | 2.5 (0-16) | 0 (0-9) | 0 (0-6) |
| *Frequency, mean (sd)* | 1.61 (1.21) | 1.92 (1.18) | 1.80 (1.08) | 1.20 (1.32) | 0.20 (0.70) |
| *Frequency, median (range)* | 2 (0-4) | 2 (0-4) | 1 (0-4) | 1 (0-3) | 0 (0-3) |
| *Intensity, mean (sd)* | 1.22 (1.07) | 1.35 (1.08) | 1.42 (0.99) | 0.90 (1.29) | 0.15 (0.49) |
| *Intensity, median (range)* | 1 (0-4) | 1 (0-4) | 1 (0-4) | 0 (0-3) | 0 (0-2) |
| 8. Required to care for more patients than I can safely care for.† |  |  |  |  |  |
| *Composite, mean (sd)* | 1.58 (2.62) | 0.48 (0.74)* | 2.61 (3.19)* | 0.80 (1.32) | 0.05 (0.22)* |
| *Composite, median (range)* | 0.5 (0-16) | 0 (0-2) | 1 (0-16) | 0 (0-4) | 0 (0-1) |
| *Frequency, mean (sd)* | 0.51 (0.74) | 0.46 (0.62) | 1.21 (0.99) | 0.70 (0.82) | 0.05 (0.22) |
| *Frequency, median (range)* | 1 (0-4) | 0 (0-2) | 1 (0-4) | 0.5 (0-2) | 0 (0-1) |
| *Intensity, mean (sd)* | 0.91 (1.09) | 0.44 (0.65) | 1.41 (1.18) | 0.50 (0.71) | 0.05 (0.22) |
| *Intensity, median (range)* | 1 (0-4) | 0 (0-2) | 1 (0-4) | 0 (0-2) | 0 (0-1) |
| 9. Experience compromised patient care due to lack of resources/equipment/bed capacity.† |  |  |  |  |  |
| *Composite, mean (sd)* | 1.73 (2.79) | 1.40 (1.94) | 2.18 (3.37)* | 2.20 (2.15) | 0.35 (0.93)* |
| *Composite, median (range)* | 1 (0-16) | 1 (0-9) | 1 (0-16) | 1.5 (0-6) | 0 (0-4) |
| *Frequency, mean (sd)* | 0.83 (0.93) | 0.94 (0.84) | 1.13 (1.01) | 1.10 (0.88) | 0.25 (0.55) |
| *Frequency, median (range)* | 1 (0-4) | 1 (0-3) | 1 (0-4) | 1 (0-2) | 0 (0-2) |
| *Intensity, mean (sd)* | 1.01 (1.07) | 0.96 (0.99) | 1.16 (1.09) | 1.40 (1.35) | 0.25 (0.55) |
| *Intensity, median (range)* | 1 (0-4) | 1 (0-3) | 1 (0-4) | 1 (0-4) | 0 (0-2) |
| 10. Required to care for patients whom I do not feel qualified to care for.† |  |  |  |  |  |
| *Composite, mean (sd)* | 0.52 (1.20) | 0.35 (0.73) | 0.61 (1.35) | 1.00 (1.83) | 0.25 (0.91) |
| *Composite, median (range)* | 0 (0-16) | 0 (0-3) | 0 (0-9) | 0.5 (0-6) | 0 (0-4) |
| *Frequency, mean (sd)* | 0.67 (0.89) | 0.31 (0.59) | 0.36 (0.62) | 0.80 (0.92) | 0.10 (0.31) |
| *Frequency, median (range)* | 0 (0-3) | 0 (0-2) | 0 (0-3) | 1 (0-3) | 0 (0-1) |
| *Intensity, mean (sd)* | 0.41 (0.77) | 0.31 (0.62) | 0.48 (0.82) | 0.60 (0.70) | 0.25 (0.91) |
| *Intensity, median (range)* | 0 (0-4) | 0 (0-3) | 0 (0-3) | 0.5 (0-2) | 0 (0-4) |
| 11. Required to work with other healthcare team members who I don't know (well). |  |  |  |  |  |
| *Composite, mean (sd)* | 1.11 (2.32) | 0.94 (1.64) | 1.25 (2.38)* | 2.30 (4.99) | 0.30 (0.73)* |
| *Composite, median (range)* | 0 (0-16) | 0 (0-9) | 1 (0-16) | 0 (0-16) | 0 (0-3) |
| *Frequency, mean (sd)* | 0.97 (0.94) | 1.25 (1.19) | 1.01 (1.06) | 1.10 (1.37) | 0.50 (0.95) |
| *Frequency, median (range)* | 1 (0-4) | 1 (0-4) | 1 (0-4) | 0.5 (0-4) | 0 (0-3) |
| *Intensity, mean (sd)* | 0.59 (0.79) | 0.50 (0.68) | 0.68 (0.78) | 0.80 (1.32) | 0.30 (0.73) |
| *Intensity, median (range)* | 0 (0-4) | 0 (0-3) | 1 (0-4) | 0 (0-4) | 0 (0-3) |
| 12. Required to work with other healthcare team members who are not as competent as patient care requires.† |  |  |  |  |  |
| *Composite, mean (sd)* | 1.14 (2.32) | 0.79 (1.41) | 1.64 (2.90)* | 0.40 (0.52) | 0.15 (0.49)* |
| *Composite, median (range)* | 0 (0-12) | 0 (0-6) | 0 (0-12) | 0 (0-1) | 0 (0-2) |
| *Frequency, mean (sd)* | 0.34 (0.62) | 0.58 (0.71) | 0.86 (1.02) | 0.40 (0.52) | 0.15 (0.37) |
| *Frequency, median (range)* | 0 (0-4) | 0 (0-3) | 1 (0-4) | 0 (0-1) | 0 (0-1) |
| *Intensity, mean (sd)* | 0.64 (0.91) | 0.56 (0.74) | 0.83 (1.04) | 0.40 (0.52) | 0.15 (0.49) |
| *Intensity, median (range)* | 0 (0-4) | 0 (0-3) | 0.5 (0-4) | 0 (0-1) | 0 (0-2) |
| 13. Witness low quality of patient care due to poor team communication.† |  |  |  |  |  |
| *Composite, mean (sd)* | 0.83 (1.94) | 0.52 (1.09) | 1.15 (2.41)* | 0.40 (0.52) | 0.35 (1.35)* |
| *Composite, median (range)* | 0 (0-12) | 0 (0-6) | 0 (0-12) | 0 (0-1) | 0 (0-6) |
| *Frequency, mean (sd)* | 1.02 (1.12) | 0.38 (0.64) | 0.67 (0.82) | 0.40 (0.52) | 0.15 (0.49) |
| *Frequency, median (range)* | 0 (0-4) | 0 (0-3) | 1 (0-4) | 0 (0-1) | 0 (0-2) |
| *Intensity, mean (sd)* | 0.58 (0.86) | 0.52 (0.77) | 0.72 (0.93) | 0.40 (0.52) | 0.20 (0.70) |
| *Intensity, median (range)* | 0 (0-4) | 0 (0-3) | 0 (0-4) | 0 (0-1) | 0 (0-3) |
| *Different compared to respondents with another professional function combined (p≤0.05), based on Mann-Whitney U test.  †Items from the Measure of Moral Distress for Healthcare Professionals. | | | | | |
