# Supplementary figures and images for: Holding the frontline: a cross-sectional survey of emergency department staff well-being and psychological distress in the course of the COVID-19 outbreak
Source: BMC Health Serv Res. 2021 May 29;21:525. doi: 10.1186/s12913-021-06555-5 (PMC8164246; doi:10.1186/s12913-021-06555-5)

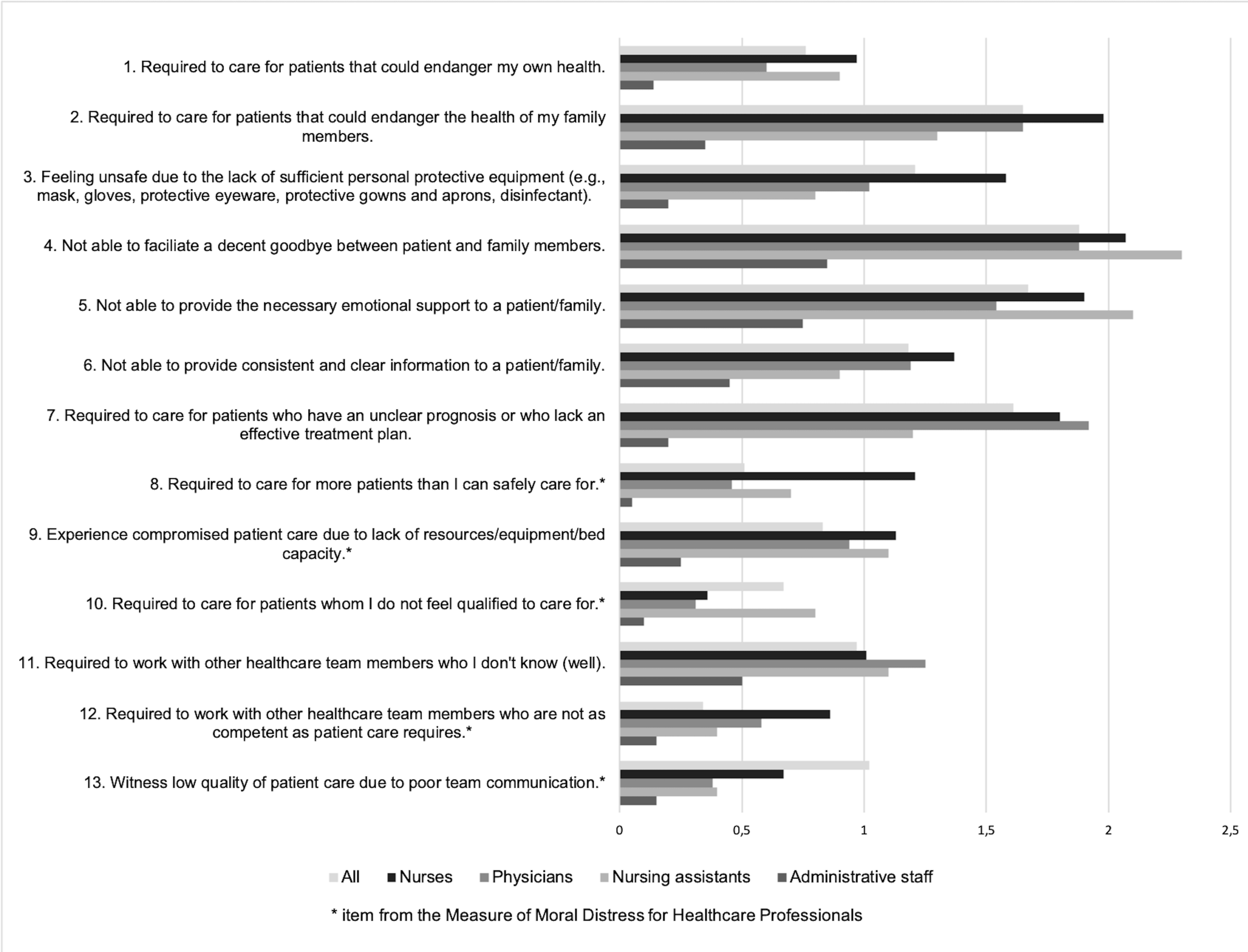

Supplement: Supplementary file 6 — Additional file 6. Figure. Mean frequency score, overall and by profession. [file 12913_2021_6555_MOESM6_ESM.tif]

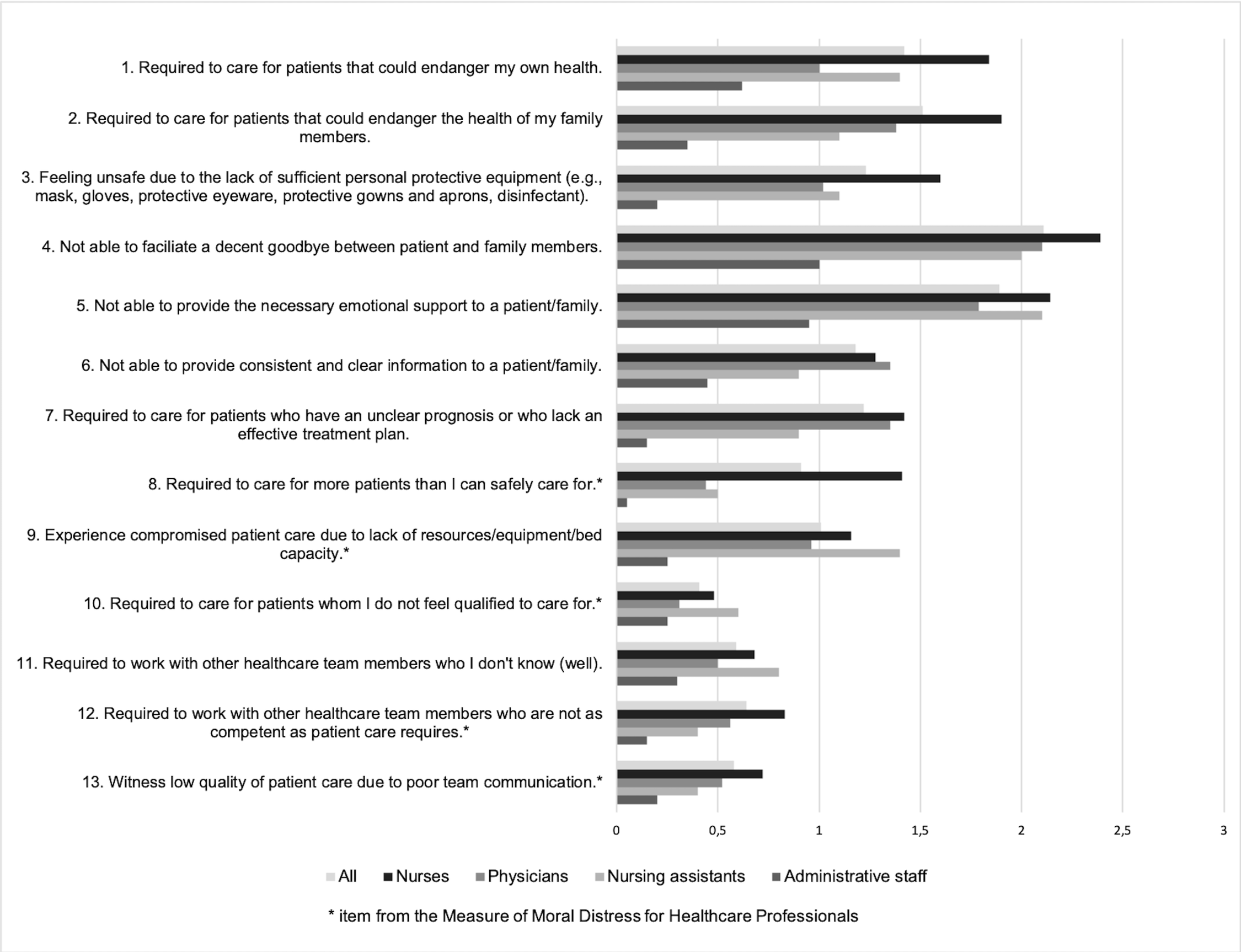

Supplement: Supplementary file 7 — Additional file 7. Figure. Mean intensity score, overall and by profession. [file 12913_2021_6555_MOESM7_ESM.tif]
